# Focus on Cdc42 in Breast Cancer: New Insights, Target Therapy Development and Non-Coding RNAs

**DOI:** 10.3390/cells8020146

**Published:** 2019-02-11

**Authors:** Yu Zhang, Jun Li, Xing-Ning Lai, Xue-Qiao Jiao, Jun-Ping Xiong, Li-Xia Xiong

**Affiliations:** Department of Pathophysiology, Jiangxi Province Key Laboratory of Tumor Pathogenesis and Molecular Pathology, Medical College, Nanchang University, 461 Bayi Road, Nanchang 330006, China; yuzhang0512@foxmail.com (Y.Z.); lj012729@163.com (J.L.); laixingning99@outlook.com (X.-N.L.); jiaoxueqiao1550@163.com (X.-Q.J.); xjp13879186822@126.com (J.-P.X.)

**Keywords:** breast cancer, Cdc42, cytoskeleton remodeling, tumor progression, targeted therapy, non-coding RNAs

## Abstract

Breast cancer is the most common malignant tumors in females. Although the conventional treatment has demonstrated a certain effect, some limitations still exist. The Rho guanosine triphosphatase (GTPase) Cdc42 (Cell division control protein 42 homolog) is often upregulated by some cell surface receptors and oncogenes in breast cancer. Cdc42 switches from inactive guanosine diphosphate (GDP)-bound to active GTP-bound though guanine-nucleotide-exchange factors (GEFs), results in activation of signaling cascades that regulate various cellular processes such as cytoskeletal changes, proliferation and polarity establishment. Targeting Cdc42 also provides a strategy for precise breast cancer therapy. In addition, Cdc42 is a potential target for several types of non-coding RNAs including microRNAs and lncRNAs. These non-coding RNAs is extensively involved in Cdc42-induced tumor processes, while many of them are aberrantly expressed. Here, we focus on the role of Cdc42 in cell morphogenesis, proliferation, motility, angiogenesis and survival, introduce the Cdc42-targeted non-coding RNAs, as well as present current development of effective Cdc42-targeted inhibitors in breast cancer.

## 1. Introduction

Breast cancer, by far the most common form of malignant tumor in females, has resulted in a steady increase in morbidity in recent decades. Even with early stage diagnosis and treatment, many patients suffer postoperative recurrence after several years. Relapse of breast cancer becomes the leading cause of death and develops in metastatic niches in bone, lung, brain, liver and other tissues through lymphatic and hematogenous vessels.

Breast cancer develops through a complicated cascade involving tumorigenesis, increased motility, cell survival and colonization. Interactions between cancer cells and their surrounding microenvironment are also required for tumor progression. Substantial evidence indicates an important role for Rho GTPase Cdc42 (Cell division control protein 42 homolog), a highly conservative protein, in the progression of breast cancer. Cdc42 deregulation is reflected in many aspects of breast cancer processes where its role seems to be highly context dependent.

## 2. Overview of Cdc42

Cdc42 is a small G protein of the Rho GTPase family. It acts as a molecular switch cycling between inactive GDP-bound and active GTP-bound states. It was initially discovered in the actin skeleton of *Saccharomyces cerevisiae* as an essential protein, which is highly conserved in human, indicating that Cdc42 may play a fundamental role in mammalian cell biology. 

Tight control of Cdc42 activation is crucial. Three protein groups; GTPase-activating proteins (GAPs), guanine-nucleotide-exchange factors (GEFs) and guanine nucleotide dissociation inhibitors (GDIs), have been found to regulate the active status of Cdc42. GAPs transform Cdc42 into an inactive GDP-bound form by raising its GTPase activity, while GEFs change GDP into GTP resulting in active GTP-bound Cdc42. GDIs are thought to sequester Cdc42 in an inactive GDP-bound state. 

Although the expression of Cdc42 is upregulated (Table 1) during breast cancer, it is not always mutated (approximately 0.1–1.7%) [1,2,3]. In fact, overexpression of Cdc42 in breast cancer is mainly mediated by cell surface receptors (such as epidermal growth factor receptor (EGFR)) or some oncogenes [4,5,6]. These factors activate Cdc42–GEFs and lead to Cdc42 hyper-activation. As a result, the deregulation of Cdc42 activates pro-tumor processes, thus affecting many aspects of breast cancer. A myriad of downstream effectors including PAKs (p21 activated kinase and all Group 1 PAKs in this review), MLK (mixed-lineage kinase) and scaffolding proteins like WASP/N-WASP (Wiskott–Aldrich syndrome protein), partitioning-defective 6 (Par6) and the IQ motif containing GTPase-activating protein (IQGAP) interact with Cdc42 to regulate these processes. Other Rho GTPases family proteins like Rac1 and RhoA can achieve a “crosstalk” with Cdc42 when necessary. In addition, Cdc42 regulation via microRNAs provides new insights and potential approaches for breast cancer treatment.

This review focuses on some important aspects of breast cancer processes and discusses the association between Rho GTPase, Cdc42 and breast cancer.

## 3. Cdc42 in Mammary Epithelial Cells Morphogenesis

Postnatal development of mammary glands is a complex process. It always begins at three weeks of age with increasing hormone levels that stimulate terminal end buds (TEBs). Concerted with mammary epithelial cells (MECs) that express receptors for progesterone and estrogen, TEBs move into the fat pad and give rise to a branched ductal tree (cap cells of TEBs give rise to basal cells while body cells form luminal cells) [26]. 

### 3.1. Cdc42 Is Essential for MECs Morphogenesis

During the early stages of development, the precise regulation of Cdc42 is crucial for normal MECs morphogenesis [27]. When Cdc42 expression is lost, MECs form significantly fewer and smaller acini that lack lumens. This disorder in acinus formation is mainly due to a break in the balance during MECs proliferation and apoptosis. Cdc42 deficiency disrupts several physiological behaviors in MECs and acini, such as cell cycle progression, mitotic spindle orientation and polarity establishment. 

In terms of the cell cycle, cyclin D1 overexpression is closely related to an increased proliferation rate in both transformed and non-transformed MECs, in vivo and in vitro [28,29]. Activated Cdc42 can stimulate cyclin D1 expression and then trigger on the G1/S transition. In Cdc42-deficient MECs, the G1/S transition is blocked resulting in a decreased proliferation rate with defective acini and a decrease in the level of pHH3—a mitosis marker [30,31]. However, the small acinus size cannot be restored by cyclin D1 overexpression, suggesting that Cdc42 is also important in other situations of cell cycle progression, in addition to G1/S [30]. Cdc42 can also promote the G2/M phase by activating PAK, whose suppression is known to result in G2/M arrest [32].

On the other hand, Cdc42 together with Par and protein kinase c (PKC), makes up a polarity complex. Establishing the Par/PKC/Cdc42 complex contributes to the apical polarity and adherens junction formation in MECs [27]. MECs-specific Cdc42-knockout can lead to the mis-localization of PKC and as a result disruption of the complex [30]. This disrupted complex not only fails to form typical apical polarity but also causes alterations in mitotic spindle orientation in MECs during 3-Dimensions cultures. Thus, it is believed that the altered mitotic spindle orientation and abnormal apical polarity contribute to the defective lumen formation in MECs acini [33,34,35]. During Cdc42 deficiency, the acini also failed to maintain the basal integrin attachment to extracellular matrix proteins (ECM), demonstrated by the mis-localization of the basal polarity marker α6-integrin [30].

### 3.2. Deregulation of Cdc42 in Breast Cancer during MECs Morphogenesis

TEBs are the most immature duct structure in the mammary gland and divide at a high rate to drive their invasion into fat pad, which make them more susceptible to cancerization [26]. Cdc42 activity in MECs is precisely controlled by multiple mechanisms, such as being maintained by RhoGDI1 [36]. Once the limitations on Cdc42 activity are released, cell cycle progression and polarity disorders can proceed to a malignant cell fate. Cdc42 overexpression can disrupt normal TEB morphogenesis and result in aberrant hyperbranching in association with stromal alterations [37]. Intriguingly, hyperactivated Cdc42-derived hyperbranching does not display a pro-proliferation phenotype but an increased intracellular contractility and cell motility phenotype that may be induced by mitogen-activated kinase (MAPK) signaling in MECs [37,38]. Moreover, stromal alterations driven by Cdc42 overexpression also result in increased ECM remodeling and stromal deposition, which also affect Cdc42 activity in MECs [39,40].

## 4. Cdc42 and Breast Cancer Cell Proliferation

### 4.1. Cdc42 Regulates Breast Cancer Cell Proliferation through MAPK Signaling

**Figure 1:**
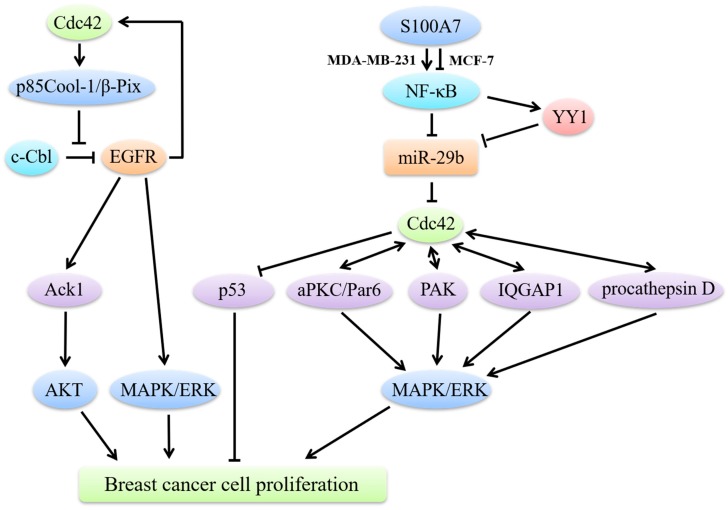
Cdc42 regulates breast cancer cell proliferation. Hyperactivated Cdc42 through p85Cool-1/β-Pix impedes c-Cbl binding to EGFR, results in EGFRs escaping from catalyzing receptor ubiquitination. Through EGFR signaling, Cdc42 induces overexpression of Ack1. Constitutive activation of Ack1 can recruit AKT to the cell membrane and subsequently activate AKT to promote breast cancer progression. A positive feedback loop exists between EGFR and Cdc42 and that EGFR is able to stimulate Cdc42 activation. Cdc42 can also interact with aPKC, overexpressed Par6, PAK, IQGAP1 and procathepsin D to promote breast cancer cell growth in a MAPK/ERK-dependent manner. Besides, Cdc42 induces p53 ubiquitination to overcome cell growth inhibition. In ER-positive MCF-7 breast cancer cells, S100A7 inhibits NF-κB. In ER-negative MDA-MB-231 cells, S100A7 activates NF-κB. NF-κB can decrease the levels of miR-29b directly or through YY1. Decreased miR-29b cannot inhibit Cdc42/p53 signaling, thus to promote breast cancer cells proliferation.

An important characteristic of carcinogenesis is malignant proliferation (Figure 1). EGF and its receptor EGFR, are the most vital factors during the proliferation process. EGFRs precisely regulate cell growth under normal conditions, while they exist in excessive amounts in breast cancer cells. The mechanisms underlying EGFR overexpression are quite complex and cannot be simply ascribed to gene amplification [41]. Binding of EGF and EGFR mainly activates the classical MAPK pathway and finally phosphorylates extracellular regulated protein kinase (ERK) to promote breast cancer cell proliferation.

Cdc42 mainly functions as an EGFR-signaling regulator in breast cancer cell proliferation. The termination of EGFR signaling requires the ubiquitin ligase activity of c-Cbl, which triggers EGFR ubiquitination and subsequent degradation. However, c-Cbl is often compromised in breast cancer and the upregulation of Cdc42 activity is considered to impair c-Cbl activation, thus inhibiting EGFR degradation [42]. It is noteworthy to mention that a positive feedback loop exists between EGFR and Cdc42 and that EGFR is able to stimulate Cdc42 activation [43]. Hyperactivated Cdc42 through its effector p85Cool-1 (cloned-out-of-library)/β-Pix (PAK-interactive exchange factor), directly impedes c-Cbl binding to EGFR, which results in EGFRs escape from catalyzing receptor ubiquitination [44]. Moreover, diabetes mellitus (DM), especially type 2 diabetes, has been recently regarded as a risk factor in breast cancer, due to the fact that high blood–glucose levels can stimulate EGFR activation and then trigger the EGFR/Cdc42 positive loop [45].

Activated Cdc42-associated tyrosine kinase (Ack1) is an oncogene encoded by the human TNK2 gene. Its overexpression in cancer cells is induced by Cdc42 through EGFR signaling. Ack1 can interact with the seven in absentia homolog (SIAH) via estrogen in breast cancer. The SIAH2 gene is a target of estrogen/estrogen receptor (ER)-signaling and mediates the ubiquitylation of Ack1 [46]. Triple negative breast cancer (TNBC) lacks ER and exhibits a high level of Ack1 [47], which correlates with high proliferation, migration and colony formation. It has been reported that constitutive activation of Ack1 can trigger the recruitment of PI3K-independent protein kinase B (PKB, also known as AKT) to the cell membrane and subsequently activate AKT in breast cancer [48], which may be the underlying mechanism of Ack1-induced tumor progression.

In addition to interacting with EGFR, Cdc42 has many other means of advancing breast cancer cell growth. Par6 can cause an EGFR-independent proliferation in normal cells except for its well-known polarity establishment function [49]. Par6 is also genomically amplified and hyperactivated in both human precancerous breast lesions and advanced breast cancer [49]. It is functionally required in breast cancer for the participation of Cdc42 and atypical PKC (aPKC). Interactive with Cdc42 and aPKC, overexpressed Par6 promotes the MAPK signaling pathway and phosphorylates ERK, even in absence of EGF/EGFR binding or ligand-independent EGFR phosphorylation [49]. Cdc42 also promotes proliferation through PAK. It is quite clear that PAK is an important downstream effector of Cdc42 and that Cdc42 binds to PAK at its N-terminal Cdc42/Rac1 interactive binding (CRIB) site. Activated PAK directly stimulates anchorage-independent proliferation of breast cancer cells by phosphorylating MEK1 and ERK1/2 [50,51]. Meanwhile, the scaffold protein IQGAP1 and procathepsin D can also interact with Cdc42 to enhance breast cancer cell growth and invasion in a MEK/ERK-dependent manner [52,53].

### 4.2. Cdc42/p53 Signaling in Breast Cancer Cell Proliferation

Activated anti-oncogene p53 exhibits multiple anti-proliferative effects including apoptosis and cell cycle arrest [54]. Studies have shown that Cdc42 can induce p53 ubiquitination to overcome cell growth inhibition [55]. However, it is notable that although Cdc42 can promote the proliferation of breast cancer cells through inhibiting p53, its precise role is breast cancer type-dependent. For example, the inflammatory protein S100A7 (Psoriasin), NF-κB and anti-oncogene miR-29b together regulate Cdc42/p53 signaling in totally different ways in ER-positive and ER-negative breast cancer cells. NF-κB can directly or indirectly decrease the levels of miR-29b, by directly binding to the miR-29b promotor or through transactivating YY1, which binds to the miR-29b promotor [56,57]. In ER-positive breast cancer, S100A7 inhibits NF-κB and restores its inhibitory effect on miR-29b. Activated miR-29b, on the one hand, inhibits Cdc42/p53 signaling and directly activates p53 to exert anti-proliferative effects. However, the opposite is observed in ER-negative breast cancers. The activity of miR-29b is inhibited by overexpressed NF-κB, which is activated by S100A7 [58].

## 5. Cdc42 and Breast Cancer Cell Motility

Cell migration is a significant phenomenon both in physiological and pathological events, such as embryogenesis, inflammatory response and wound healing [59], especially in cancer metastasis. More attention needs to be paid to the relationship between Cdc42 and breast cancer cell metastasis. Overexpression of Cdc42 usually leads to cancer cell migration and invasion, which are required for breast cancer spreading into the surrounding tissues and its distant metastasis. In these multifaceted processes, polarized cells extend motile protrusions (characterized as lamellipodia, filopodia and invadopodia) in the cell front to interact with the ECM and neighboring cells, then contract the cell body and detach the cell rear from the matrix to move forward [1]. It is a cyclic process initiated with the cells response to polarize and extend protrusions in the direction of migration [59]. During this process, Cdc42 can function as a central regulator via controlling the reorganization of the actin-based cytoskeleton and cell-cell junctions.

### 5.1. Cdc42 Is a Key Regulator of Migratory Protrusion Formation

Breast cancer can move via single cells or collective clusters and both movement forms have different mechanisms [60]. In the process of collective cell migration, Cdc42 is mainly involved in regulating the polymerization of actin filaments that drive protrusion formation. Cell filament geometry consists of two basic choices; branched filaments that lead to sheet-like protrusions characterized as lamellipodia and long parallel or bundled filaments that lead to spike-like filopodia [61].

The lamellipodia formation can be described as follows: Actin filaments polarize with the formation of fast-growing “barbed ends” and slow-growing “pointed ends” to drive protrusions [62]. Cofilin can sever pre-existing actin filaments to produce free barbed ends. The Src/FAK (proteins kinase/focal adhesion kinase) complex activates Paxillin (a scaffolding protein that can recruit several regulatory and structural proteins to modulate cell adhesions and cytoskeleton reorganization [63]) to recruit Cdc42 and trigger N-WASP activation. The combination of activated N-WASP with actin-related protein 2/3 (Arp2/3) leads to conformation changes of Arp2/3 and brings actin monomers (G-actin) to this complex [1]. Subsequently, the Arp2/3 complex mediates actin nucleation of new filaments at the cofilin-severed barbed ends [64,65,66] Rac can also modulate actin nucleation. Active Rac proteins can interact with a WAVE-associated complex of proteins, which in turn activates actin nucleation via Arp2/3. Furthermore, an extension of the actin filament can also be induced by Rac via interaction with scaffold protein lamellipodin that binds with WAVE complex [67,68]. Actually, the Rac-mediated mechanism is predominant during lamellipodia formation. In contrast, Cdc42 or other Rho GTPases activate formins to extend Arp2/3 complex-induced filaments. Then profilin delivers G-actin to formins to elongate the linear actin network and to facilitate rapid actin assembly [69] The renewal of such an actin network largely depends on the regulation of cofilin. Cofilin severs and depolymerizes older actin filaments in the network, leading to the rapid turnover of actin filaments [70]. Activated Rac or Cdc42 can activate PAK1 and its downstream effector LIM kinase 1 (LIMK1), which contributes to the inactivation of cofilin. A lack of its actin filament (F-actin, filamentous state of actin, can be converted from G-actin [71])-depolymerizing activity leads to the accumulation and aggregation of actin filaments [72,73]. Due to the inactivation of cofilin, rapid actin filaments turnover slows down and a relatively stable network is generated. Enhancement of stable actin filaments, in turn, reduces cell migration [74]. In breast cancer cells, the activation and inactivation of cofilin are unbalanced, altering protrusions and cell motility [75]. Vasodilator-stimulated phosphoprotein (VASP), termed as “anti-cappers,” can prevent blockage of actin filaments by capping proteins, thus promoting the formation of unbranched actin networks in lamellipodia [76]. However, silencing Cdc42 does not block the formation of lamellipodia in MDA-MB-231 cells [42]. The regulation of lamellipodia formation is predominantly dependent on the activation of Rac and Cdc42 mainly functions to modulate the formation of filopodia.

The formation of filopodia is initiated by IRSp53 (insulin receptor phosphotyrosine 53 kDa substrate, a multi-domain protein that induces filopodia through its I-BAR domain [77]). IRSp53 can bend the membrane and recruit Cdc42 and diaphanous-related formin 3 (DRF3 = mDia2), which in turn mediates actin nucleation. VASP delivers actin monomers to the filopodial tip and G-actin is provided directly to mDia2 by profilin. Cdc42 and Rif can regulate actin polymerization by targeting mDia2 and Cdc42 can stimulate N-WASP/Arp2/3-driven polymerization, similar to the mechanism in lamellipodia [69]. Filopodia is not necessary for cell migration. It is usually considered to be an environmental sensor that can also contribute to migration by converting to lamellipodia during growth factor receptor signaling [62,78].

**Figure 2:**
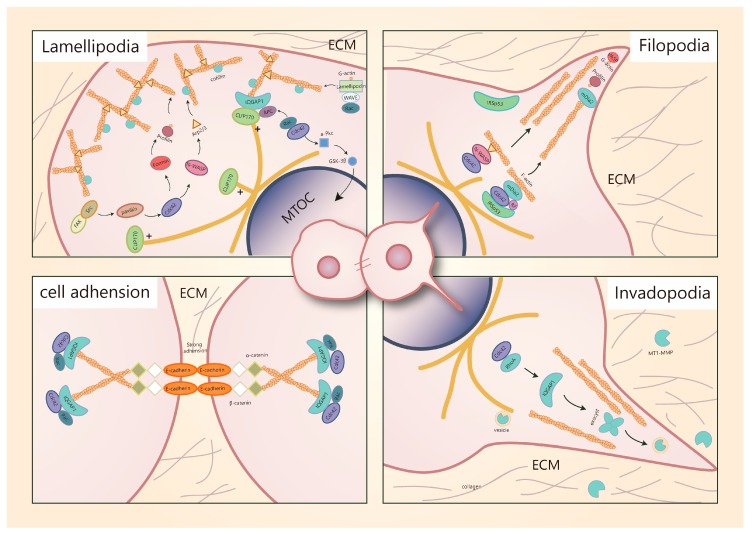
Role of Cdc42 on regulation of migratory protrusions formation. Model of lamellipodia formation: cofilin severs pre-existing actin filaments to produce free barbed ends. The Src/FAK complex activates Paxillin to recruit Cdc42 and trigger N-WASP activation. The combination of activated N-WASP with Arp2/3 leads to actin nucleation of new filaments at the cofilin-severed barbed ends. Cdc42 activates formins. Profilin delivers G-actin to formins to extend filaments. Rac interacts with lamellipodin that binds with WAVE to extend actin filaments. Model of filopodia formation: IRSp53 recruits Cdc42 and mDia2. mDia2 mediates actin nucleation. VASP delivers actin monomers to the filopodial tip and G-actin is provided directly to mDia2 by duringilin. Cdc42 and Rif can regulate actin polymerization by targeting mDia2. Cdc42 can also stimulate N-WASP/Arp2/3-driven polymerization. Model of invadopodia formation: Cdc42 and RhoA trigger the interaction of IQGAP1 with the exocyst subunits Sec3 and Sec8, which is necessary for invadopodia activity. MT1-MMP is transported to the tip of invadopodia by microtubule-mediated vesicle trafficking to degrade the extracellular matrix.

Another special protrusion called invadopodium is often assembled for cancer cell invasion. Such protrusions can secrete metalloproteases (MMPs) at the front cells to degrade extracellular matrix and basement membrane components [79]. Membrane type 1 metalloprotease (MT1-MMP) and perhaps other MMPs are transported to the tip of invadopodia by microtubule-mediated vesicle trafficking, which requires ADP ribosylation factor 6 (ARF6) [69]. There are many similarities in the regulatory mechanisms involved in the formation of filopodia, lamellipodia and invadopodia. The key difference is that invadopodia can degrade the extracellular matrix; therefore, the delivery of vesicles containing matrix-degrading proteases, in particular, MT1-MMP is required. These vesicles target invadopodia through the vesicle-tethering exocyst complex [80]. In highly invasive MDA-MB-231 human breast carcinoma cells, activated Cdc42 and RhoA can trigger the interaction of IQGAP1 with the exocyst subunits Sec3 and Sec8, which is necessary for invadopodia activity, because the deletion of the exocyst-binding site is accompanied by the loss of IQGAP1-induced enhancement of matrix degradation. Thus, the exocyst and IQGAP1 are required for the accumulation of cell surface MT1-MMP at invadopodia [81] (Figure 2).

However, the regulation of Cdc42 during pseudopods formation is not specific. Overexpression of podoplanin in MCF-7 cells induces filopodia formation and cell polarization, leading to the enhanced β1-integrin-mediated cell spreading and adhesion on the extracellular matrix, thus increasing cell migration and invasion [82]. Furthermore, Stromal cells such as fibroblasts may also induce collective cancer cell migration, playing a similar role to MMPs to open a way for trailing cells. In this model, Cdc42-mediated activation of MRCK is required to allow cancer cell migration behind leading fibroblasts [83]. Protrusions of the plasma membrane at the front of cell groups drive the movement of the clusters.

### 5.2. Cdc42 Modulates the Establishment of Cell Polarity

A polarized morphology is required to form a stable actin network, which is a prerequisite for directed cell migration. Migrating cells dynamically polarize during the process of movement.

**Figure 3:**
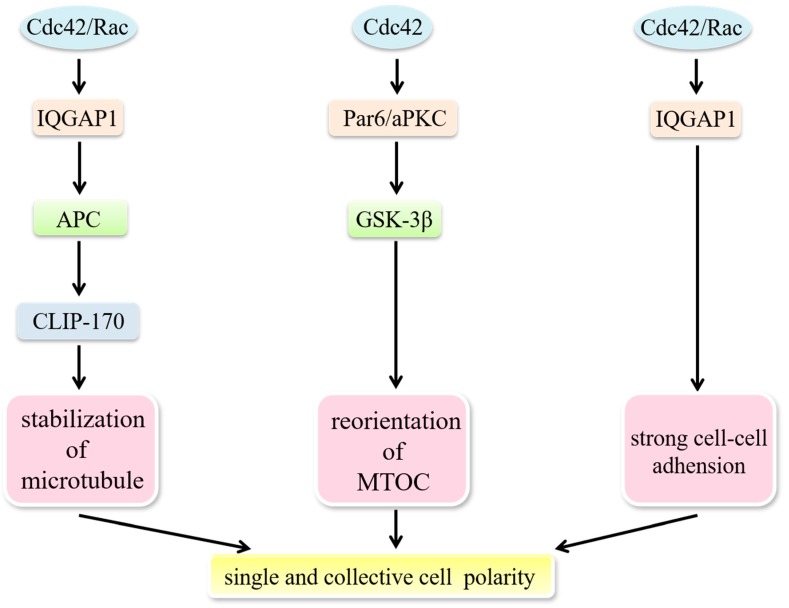
Cdc42 modulates the establishment of cell polarity. Establishment of single-cell polarity: Activated Rac1 and Cdc42 mark spots where IQGAP1 tethers actin filaments. IQGAP1 links APC to actin filaments and captures the plus-ends of microtubules through the microtubule-binding protein CLIP-170, which stabilizes microtubules and generates a stable actin meshwork at the leading edge. Cdc42 regulates the reorientation of the MTOC via a Par6/aPKC complex to induce the phosphorylation of GSK-3β. Maintenance of collective polarity: Strong cell-cell adhesion is established when the increasingly active Rac1 and Cdc42 interact with IQGAP1 to crosslink actin filaments.

Establishing polarity demands asymmetric distribution of the cytoskeleton, cell-adhesion molecules and signaling molecules, as well as directed membrane trafficking performed by motor proteins such as dynein and kinesin. The model consists of several coordinated processes, including; membrane ruffling and filopodia at the leading edge, capture of microtubule plus-ends near the leading edge and reorientation of the microtubule-organizing center (MTOC) and the Golgi apparatus towards the direction of migration [84]. Nuclear repositioning is an initial polarizing event in migrating cells. The nucleus moves away from the leading edge to reorient the MTOC, while the MTOC remains stationary, which is coupled with actin retrograde flow and is regulated by a pathway involving Cdc42, MRCK, myosin and actin [85]. Cdc42 participates in regulating the establishment of single-cell polarity through modulating microtubule-based intracellular vesicle trafficking to the apical cell surface and orientation of the cell division spindle. Furthermore, Cdc42 plays a role in maintaining collective cell polarity by strengthening cell–cell junctions [4] (Figure 3). Activated Rac1 and Cdc42 are able to mark spots where IQGAP1 tethers actin filaments. IQGAP1 then acts as a scaffold linking adenomatous polyposis coli (APC) to actin filaments and captures the plus-ends of microtubules through the microtubule-binding protein CLIP-170, which directly and/or indirectly stabilizes microtubules and generates a stable actin meshwork at the leading edge [86]. Cdc42 can also regulate the reorientation of the MTOC via a Par6–atypical protein kinase C (aPKC) complex, which induces the phosphorylation of GSK-3β and the interaction of APC with the plus ends of microtubules [87]. Microtubule-mediated delivery of vesicles and the associated proteins needed are provided to the membrane at the leading edge [62,88].

Specialized cell–cell adhesion complexes, characterized as E-cadherin-containing adherens junctions (AJs), are also necessary to help maintain proper barrier function and apical-basolateral polarity in epithelial cells. Disruption of these normal characteristics in epithelial cells has been associated with tumor progression, such as epithelial-mesenchymal transition (EMT). A dynamic equilibrium exists between the E-cadherin–β-catenin–α-catenin and E-cadherin–β-catenin–IQGAP1 complexes at sites of cell–cell contact. The ratio between these two complexes could determine adhesion strength [86]. Strong adhesion is established when the increasingly active Rac1 and Cdc42 interact with IQGAP1 to crosslink actin filaments and weak adhesion is built under the opposite conditions. This is due to the fact that free IQGAP1 interacts with β-catenin to dissociate α-catenin from the cadherin–catenin complex [86]. Such deficient adhesions facilitate EMT.

### 5.3. Cdc42 Involves the Progression of EMT

During the process of EMT, epithelial cells lose cell-to-cell interactions and cell polarity, tissue structures become loose and transform from polygonal epithelial cells to a spindle-like fibrocyte-like morphology. Moreover, apical and basolateral epithelial-specific proteins in cells such as E-cadherin, catenins and cytokeratins progressively redistribute or downregulate, while mesenchymal molecules such as vimentin, fibronectin and N-cadherin are re-expressed. These series of changes confer breast cancer cells with the motility necessary for invasion [82]. It is noteworthy that EMT does not occur in the case of collective cell migration. Interferon regulatory factor 4 binding protein (IBP, a Rho-family guanine nucleotide exchange factor for Rho family GTPases, including Rac1, RhoA and Cdc42 [89]) can mediate Rac1, RhoA and Cdc42 activation in breast cancer cells to regulate actin cytoskeleton rearrangement and MMP production. Meanwhile, IBP also decreases the expression of the epithelial markers E-cadherin and keratin 18 but increases the expression of mesenchymal markers fibronectin and N-cadherin to trigger the acquisition of an EMT phenotype [90]. Consequently, IBP may regulate EMT and the movement of breast cancer cells via Rac1, RhoA and Cdc42 signaling pathways.

### 5.4. Cdc42 Regulates Breast Cancer Cells Motility via Various Effectors

Various regulators have been reported to target Cdc42 and influence breast cancer movement due to Cdc42 functions. In T47D mammary epithelial cells, activation of PI3K via chronic activation of Cdc42 and Rac1 disrupts the normal, polarized organization of these cells and promotes a motile, invasive phenotype [91]. Melanoma differentiation-associated gene-9 (MDA-9), also known as syntenin-1 (SDCBP; syndecan binding protein), a member of the PDZ-domain-containing family [92], modulates the small Rho GTPases RhoA and Cdc42 to enhance invasion and cytoskeletal rearrangement in MDA-MB-231 and SUM159 breast cancer cells via TGFβ1 [93]. The Kruppel-like factor 5 (KLF5) transcription factor, highly expressed in high-grade, poorly differentiated and basal-like triple-negative breast cancer (TNBC [94]), directly binds to the *TNFAIP2* gene promoter and activates its transcription. *TNFAIP2* then interacts with Rac1 and Cdc42, increases their activities to change the actin cytoskeleton and cell morphology, thus promoting TNBC cells migration and invasion [95]. A recent study demonstrated a novel ability of Cdc42 to modulate cell migration in MDA-MB-231 and Hs578T cells. ERK5, also known as big MAP kinase 1 (BMK1), a member of MAPK family [96], can decrease the migration and invasion of both MDA-MB-231 and Hs578T cells. Cdc42 has been shown to inhibit its phosphorylation and expression to increase cell motility [97]. In MCF-7 and MDA-231 cells, δ-Catenin (a member of the P120 catenin (p120ctn) family [98]) upregulates Cdc42 and Rac1 activities and contributes to increased cell mobility [99]. Invasion of MDA-MB-231 cells into three-dimensional (3-D) type I-collagen matrices depends on TGF-α. This event is likely dependent on the activation of Cdc42 via TGF-α to initiate the formation of protrusions into collagen [100]. P120 catenin (p120), a Src substrate that can indirectly activate Rac1 and Cdc42, acts as an obligate intermediate between ErbB2 and Rac1/Cdc42 to modulate the metastatic potential of breast cancer cells [101,102]. To summarize, Cdc42 acts as a significant regulator in breast cancer cell migration and invasion.

## 6. Cdc42 and Breast Cancer Angiogenesis

The rapid growth of breast cancer cells depends on the constant supply of nutrients by blood vessel networks but the intrinsic vascular network cannot provide such large amounts of nutrients. As a result, breast cancer cell progression requires newly expanding blood vessels [103]. Angiogenesis is the process of new blood vessels arising from existing vessels, which requires vascular endothelial cell proliferation and migration as well as basement membrane breakdown. This process is accurately controlled by many pro-angiogenic factors including EGF, fibroblast growth factors (FGF), vascular endothelial growth factor (VEGF), IL-6 and IL-8, in addition to anti-angiogenic factors including angiostatin [104]. While these pro- and anti-angiogenic factors are in a dynamic balance under normal condition, during breast cancer the balance is tipped and pro-angiogenic activity dominates.

The basic mechanisms of Cdc42 regulating vascular endothelial cell proliferation, migration and basement membrane breakdown are the same as those mentioned previously. These do not however describe the entire role of Cdc42, which can also particularly regulate pro-angiogenic factors during breast cancer angiogenesis. Since its definition in the 1980s, VEGF has been the most important pro-angiogenic factor. It is overexpressed in a broad spectrum of cancers and considered as the major initiator of pathological angiogenesis [105]. High expression of VEGF often occurs in ischemic areas of tumors, which is induced by hypoxia, which also activates Cdc42 through PI3K and PTK [106]. It has been reported that hyperactivated Cdc42 (under both normoxic and hypoxic conditions) upregulates VEGF in breast cancer cells [106]. Cdc42 does not regulate VEGF directly but through p53. Mammary VEGF transcription is inhibited by p53 in many ways. Firstly, the VEGF promoter contains specificity protein-1 (Sp1) binding sites [107], where p53 forms complexes with Sp1 to prevent the VEGF transcription [108]. p53 can also regulate hypoxia-inducible factor-1 (HIF-1α) and proto-oncogene *c*-*Src* activity, thus decreasing VEGF mRNA transcription under hypoxic condition [108,109]. Cdc42 participates in VEGF-mediated angiogenesis mainly by degrading p53 to relieve VEGF inhibition [55]. Furthermore, hypoxia-activated Cdc42 can also increase the levels of IL-6 and IL-8 to upregulate VEGF expression [110,111], which is achieved by Cdc42 activating NF-κB, a modulator of IL-6/8 expression [112,113]. In addition to VEGF, FGF is another strong pro-angiogenic factor overexpressed in breast cancer [114,115,116]. Cdc42 can bind to FGF1 promotor at Ets sites, leading to increased transcription [117].

## 7. Survival of Breast Cancer Cells Requires Cdc42

The human body itself, after it becomes aware of malignant proliferation and breast cancer cells invasion, initiates a series of responses such as apoptosis and immune responses to prevent unlimited cancer cell growth. Anti-cancer drugs (chemotherapy) have also been used as effective treatments to eliminate cancer cells and prolong patient survival. However, Cdc42 assists breast cancer cells in escaping apoptosis and chemotherapeutic treatments, allowing them to survive in circulation.

Cellular apoptosis in the human body is driven by many apoptosis-related genes like members of the *Bcl*-*2* family, anti-oncogene *p53*, proto-oncogene *c*-*Myc* and *Fas*. It is also mediated by immune cells like T cells and natural killer cells (NK cells). Cdc42-mediated anti-apoptosis consists of many aspects, including its interactions with some of these apoptosis-related genes and immune cells as well as a “crosstalk” with other Rho GTPases.

### 7.1. Cdc42 Regulates Apoptosis-Related Genes through PAK and JNK Signaling

The *Bcl*-*2* family consists of cell death genes (*Bad*, *Bax*, *Bak* and *Bcl*-*x_S_*) and cell survival genes (*Bcl*-*2*, *Bcl*-*x_L_*, *Mcl*-*1* and *A1*) [118]. These genes are critical to intrinsic cell death machinery and relative levels of them dictate the susceptibility of cell death [119]. One of the important ways Cdc42 affect *Bcl*-*2* family during breast cancer is by stimulating its downstream effector PAK (both PAK1 and PAK2). PAK is capable of phosphorylating the pro-apoptotic member Bad on both Ser112 and Ser136 to reduce the interaction between Bad and the cell survival members Bcl-2 and Bcl-x_L_ [120]. Dissociation of the Bad/Bcl-2/Bcl-x_L_ complex ultimately results in an inhibition of mitochondrial cytochrome c, thus suppressing cell death [121]. Moreover, PAK also activates NF-κB by stimulating the p65 subunits nuclear translocation to prevent apoptosis of breast cancer cells [122,123].

Using alternative mechanisms to ERK, c-Jun N-terminal kinase (JNK) is another branch of the MAPK signaling pathway, which regulates apoptosis in breast cancer [124]. Constitutive activation of Cdc42 can activate JNK through MKK4/7, which then activates an important transcription factor AP-1 [125,126]. In humans, AP-1 constitutes two subunits, jun and fos [127]. These two subunits act quite differently in the transcription of many apoptosis-related target genes and trigger different roles in breast cancer apoptosis. Jun is also a regulator of Bcl-2 family members but its major effect is to downregulate the transcription of *Bcl-2* and *Bcl-xL*, which is distinguished from Cdc42/PAK pathway [128]. Without jun, Bcl-2 and Bcl-x_L_ can also be phosphorylated directly by JNK [129,130]. Besides, Jun is involved in the regulation of anti-oncogene *p53* as well, inhibiting p53 transcription to resist apoptosis and promote proliferation [131]. Fas/FasL-induced death receptor pathway is another crucial apoptotic mechanism in addition to the mitochondrial pathway that is induced by the *Bcl*-*2* family. Jun together with fos or STAT3, is reported to regulate Fas/FasL expression [132,133]. Fas-induced apoptosis is also dependent on caspases to cleave substrates that are important for cell survival. In breast cancer, caspase-3/7 can cleave its key substrate protein Cdc42 at aspartate acid residues 121 and 118. However, the expression of mutated caspase-insensitive Cdc42 slows down the Fas-induced apoptotic response and displays a strong anti-apoptotic effect [43]. This Cdc42 mutant may exist in breast cancer allowing it to overcome Fas-induced apoptosis. FasL has been shown to elevate a part of the Cdc42 pool but some cascade amplification is still required to affect the Fas–caspase system [43].

### 7.2. Cdc42 Drives Actin Responses in NK Cells

NK cells are large granular lymphocytes in morphology with cytotoxic activity against virus-infected cells and cancer cells. The immunological synapse (IS) is an indispensable structure between NK and target cells, required for the recruitment and release of intercellular lytic granule to the target cell. A significant phenomenon when breast cancer cells respond to NK cells is a massive and rapid F-actin accumulation surrounding IS, termed “actin response,” which is responsible for NK cell resistance. This burst actin response is mainly induced by Cdc42/N-WASP signaling along with their downstream Arp2/3 complex. Inhibition of Cdc42/N-WASP significantly increases the levels of cytotoxic protease granzyme B in target cells and is sufficient to transform NK cell-resistant breast cancer cells into susceptible ones [134].

### 7.3. Crosstalk of RhoGTPases during Breast Cancer Apoptosis

Cdc42 participates in a “crosstalk” with Rho GTPases for an anti-apoptotic function. Cdc42 has long been known to activate Rac1, which leads to RhoA activation. The anti-apoptotic role of RhoA includes inhibiting the cell cycle inhibitor p21 to enhance cell survival and activating *Bcl*-*2* family members [135,136]. Injection of RhoA or Cdc42 prevents breast cancer cells from mAb200 (Ras-GAP inhibitor)-induced apoptosis but no additional effects are seen upon Cdc42/RhoA co-injection, which demonstrates that the protective function of Cdc42 in breast cancer results from RhoA activation [137].

### 7.4. Cdc42 and Anti-Cancer Drugs Resistance

Breast cancer is sensitive to chemotherapy and adjuvant chemotherapy in later stages of treatment. However, multidrug resistance (MDR) remains an important cause of chemotherapy failure and clinical treatment disturbance [138]. Cancer cells activate the transcription of drug-resistant genes through various signaling pathways, leading to an increased expression of drug-resistant proteins and eventually drug resistance. Cdc42 is one of these drug-resistant proteins involved in breast cancer.

Doxorubicin (Adriamycin, ADM) is a broad-spectrum anti-cancer drug, which can target breast cancer. Its mechanism of action involves inhibiting the synthesis of nucleic acids by intercalating DNA [139]. In ADM-resistant breast cancer cells, transfection of Cdc42-specific siRNA can significantly increase ADM levels and enhance its killing effects on these ADM-resistant cells [140]. Moreover, breast cancer is a hormone-dependent systemic disease and many of its processes are related to estrogen [141,142]. After estradiol-17 beta treatment, breast cancer cells express higher Cdc42 levels and exhibit stronger ADM resistance, which is directly manifested by the decrease chemotherapeutic drug accumulation in cells [143]. It is suggested that Cdc42 participates in anti-cancer drug resistance by interacting with N-WASP and Arp2/3 to promote actin polymerization, microfilament cytoskeleton rearrangement, intracellular material flow acceleration and promotion of intracellular drug excretion to the extracellular space.

Besides phosphorylating *Bcl-2* family members to protect breast cancer cells from intrinsic cell death, Cdc42 downstream effector PAK also regulates anti-cancer drugs-induced cell death. It has been demonstrated that breast cancer cells with low PAK2 activity exhibit strong sensitivity to the anti-cancer drugs cisplatin and taxol [144]. PAK2 is unique among the PAK family: Activated by Cdc42, full-length PAK2 protects breast cancer cells from drug-induced cell death; when proteolytically cleaved by caspase 3, PAK2 generates its fragment PAK2p34 that favors apoptosis [145]. PAK2 downregulates caspase 3 to block the generation of PAK2p34 and promotes breast cancer cell survival, leading to MDR.

## 8. Current Research Advances of Cdc42-Targeted Therapies in Breast Cancer

The contribution of Cdc42 to breast cancer cells is substantial, due to its critical roles in many aspects of cancer processes. However, drugs targeting Cdc42 were once considered impossible due to its micromolar levels in cells and its perplexing signal transduction with other factors. Nonetheless, in recent years, some Cdc42-targeted drugs are being developed in breast cancer research, aiming to inhibit Cdc42 activation in various ways (Table 2).

### 8.1. GEF Interaction Inhibitors

GEFs exchange GDP into GTP and generate active-bound Rho GTPases. NSC23766 is designed on the Trp56 residue of Rac, which is vital for GEF binding [146]. However, off-target effects prevent clinical use of this drug. EHop-016 is another Rac inhibitor derived from NSC23766, also targeting Trp56 [147]. In metastatic cancer cells, EHop-016 inhibits Cdc42 activation with an IC_50_ approximately >10 μmol/L [148]. Moreover, EHop-016 has the capacity to inhibit breast cancer cell growth (approximately 80%) [148] and block angiogenesis and metastasis [149]. However, its bioavailability and high effective concentrations need to be improved [150].

In recent years, MBQ-167 has been designed to form H bonds with the Asn39 side-chain of Cdc42 and Rac and Asn39 replacement leads to loss of GEF binding [146]. Surprisingly, MBQ-167 inhibits Cdc42 activation with an IC_50_ of 78 nmol/L, which make it one of the most effective Cdc42 inhibitors at present [151,152]. MBQ-167 has been shown to inhibit a large proportion of Cdc42 downstream effectors, like PAK and LIMK. Interestingly, the PAK1 displays autophosphorylation when MBQ-167 inhibits Cdc42, suggesting a feedback loop in MBQ-167/PAK1. Nevertheless, expression of PAK effectors LIMK and cofilin are significantly blocked by MBQ-167, which inhibits PAK1 [153,154].

MBQ-167 can inhibit nearly all Cdc42-induced tumor processes in breast cancer, including cell polarity, cell cycle progression, apoptosis and metastasis [152]. However, MBQ-167 only inhibits Cdc42 activity in breast cancer cells that undergo EMT, rather than non-cancer cells or cancer cells without EMT [151]. This selective inhibition of MBQ-167 may result from the different Cdc42-related GEF expression profiles in different types of breast cancer; MBQ-167 may only affect a subset of Cdc42-related GEFs that are activated in mesenchymal-like breast cancer cells [155]. Focal adhesion assembly at the mesenchymal-like breast cancer cell leading edge is regulated by integrins that are under Cdc42 regulation, while the integrins that regulate mammary epithelial cell filament cytoskeleton are not directly mediated by Cdc42 [156,157]. Based on this selectivity, MBQ-167 can reduce the viability of breast cancer cells with EMT process instead of non-cancer cells, which makes MBQ-167 more tumor-specific. Since EMT is also related to drug resistance [158], MBQ-167 has the potential to prevent drug resistance. In nude mice mammary fat pad tumors, the use of MBQ-167 reduces tumor size by about 91% in two months with 10 mg/kg bodyweight (BW) and no metastases are observed [151].

### 8.2. Nucleotide Binding Inhibitors

Aside from preventing GEFs binding to Cdc42, an alternative to Cdc42 targeting is to block nucleotide binding. R-enantiomer of ketorolac (R-ketorolac), the allosteric inhibitor of Cdc42 and Rac, is the first FDA-approved Cdc42 and Rac inhibitor proceeding to P0 clinical trials [159]. It is reported to inhibit tumor progression in breast cancer virus-polyoma middle T antigen (MMTV-PyMT) mice [160].

The topoisomerase II inhibitor, mitoxantrone (MTX), is also an FDA-approved drug in breast cancer. MTX can block GTP binding of Cdc42, then inhibit actin filament cytoskeleton and reduce cell migration [161].

### 8.3. RhoGDI Modulators

RhoGDIs are thought to sequester Cdc42 in the inactive GDP-bound state within the cytosol. The design that prevents the dissociation of RhoGDIs and Cdc42 is a potential strategy for Cdc42-targeted treatment. Secramine has been demonstrated to inhibit Cdc42 activation in a RhoGDI-dependent manner. More specifically, secramine inhibits the PIP2-stimulated Cdc42/N-WASP/Arp2/3-mediated actin polymerization and this inhibitory effect requires the presence of RhoGDI1 to prevent the membrane recruitment of Cdc42 [162]. However, the secramine-induced inhibition is not selective and both RhoGDIs upregulation and downregulation are reported with increasing malignancy [163].

### 8.4. Metformin

TNBC refers to breast cancers whose immunohistochemical results are ER-negative, progesterone receptor (PR)-negative and HER2-negative. This kind of breast cancer lacks specific clinical therapeutic guidelines [164]. Luckily, the antidiabetic drug metformin has been reported to inhibit breast cancer cell proliferation and migration by significantly downregulating Cdc42 expression and TNBC is sensitive to metformin [165,166]. Metformin is thought to exhibit anti-cancer activity via the AMP-activated protein kinase (AMPK) signaling pathway [167]. In this signaling network, an increased level of AMP activates AMPK, which inhibits the mammalian target of rapamycin (mTOR) expression to reduce tumor progression [167]. However, metformin-mediated Cdc42 downregulation does not require this typical AMPK signaling pathway; conversely, AMPK upregulates Cdc42 expression [168]. Downregulation of Cdc42, induced by metformin, is partially due to transcription factors such as DNTTIP2, TCEB2 and YWHAB [168].

### 8.5. Biological Extractions

A few advances have been made in traditional Chinese medicine-related anti-cancer treatments in recent years. Cdc42 is an effective target for traditional Chinese medicine which has been used to inhibit breast cancer progression. A traditional Chinese medical herb, *Ganoderma lucidum* (GA), has been demonstrated to reduce breast cancer cell proliferation and migration, as well as induce apoptosis [169,170]. *Ganoderma lucidum* triterpenoids extracts (GAEE) that contain GA, GA isomer and dehydrogenated GA, can reduce FAK activation and break the interaction between FAK and Src, then attenuate the affinity between the Src/FAK complex and Paxillin in breast cancer. The Cdc42 recruitment function of Paxillin allows GAEE to downregulate Cdc42 expression and attenuates the interaction between Cdc42 and N-WASP, which results in an impairment at the cell leading edge, thus inhibiting cell migration [171]. Therefore, GAEE may be a potent anti-cancer drug in breast cancer.

Resveratrol (trans-3,4V,5-trihydroxystilbene) is a phytoalexin that was initially extracted from grapes. It can bind to ER and display opposing effects, for example, it is estrogenic at low concentrations and anti-estrogenic at high concentrations [142,172]. High concentration resveratrol-induced inhibition of Cdc42 results in a widespread and sustained filopodia response, which is due to the inhibition of Rac activation. Rac converts filopodia to lamellipodia [78], while reduced Rac activation ultimately leads to the occurrence of non-polar filopodia, which inhibits the migration of breast cancer cells [142,172].

## 9. Cdc42-Related Non-Coding RNAs in Breast Cancer

### 9.1. microRNA

MicroRNA is a type of endogenous small non-coding RNA (ncRNA) with a length of about 20–24 nucleotides. Each microRNA can have multiple target genes and several microRNAs can also regulate the same gene [173]. MicroRNAs decrease the expression of target genes by forming a complement with the mRNAs of their target genes [174]. The 3′-untranslated region (UTR) is the crucial site for the regulation of microRNA functions [175]. Due to their important regulatory roles in cells, deregulation of microRNAs always occurs in many diseases including cancers. In breast cancers, some microRNAs target Cdc42 and are extensively involved in Cdc42-induced tumor processes, while many are aberrantly expressed (Table 3).

As mentioned previously, miR-29b regulates Cdc42/p53 signaling during breast cancer cell proliferation. Another miR-29 family member, miR-29a, which also targets Cdc42 is downregulated in breast cancer. It is identified as a tumor suppressor due to its inhibitory regulation of Cdc42 during cell cycle progression [176].

Compared to regulating cell growth, microRNAs interfere more in actin cytoskeleton regulation functions of Cdc42. miR-206 has been demonstrated to regulate actin remodeling during breast cancer metastasis and Cdc42 is a potential target of miR-206. During metastasis, miR-206 can inhibit filopodia formation and matrix degradation by inhibiting Cdc42 activation [177]. miR-23b also has a vital role in the actin cytoskeleton [178]. PAK is known to restrict the size of focal adhesions (focal adhesions that mature excessively are related to slower migration rates) to promote migration [179]. miR-23b downregulates the Rac/Cdc42 guanine nucleotide exchange factor 6 (ARHGEF6) which activates Cdc42/PAK, thus enhancing focal adhesions maturation in breast cancer [180]. In addition, overexpression of miR-23b is associated with an increasing epithelial phenotype in breast cancer cells, which leads to the EMT inhibition function of miR-23b [180]. Moreover, miR-224 can inhibit breast cancer cell invasion by directly suppressing Cdc42 during the interaction at their binding site [181]. miR-888 also inhibits the adherens junction (AJ) pathway by targeting Cdc42 [182]. Furthermore, CD44 3′-UTR has a decoy effect that binds to miR-216a, miR-330 and miR-608 resulting in increased Cdc42 expression in MT-1 breast-carcinoma cells [175].

Hyperactivation of the cancer stem cell (CSC) pool in breast cancer patients with hyperglycemia is associated with miR-424 regulation of Cdc42. miR-424 interacts with the Cdc42 promoter sequence through the complementarity between them. The ectopic expression of miR-424, which always occurs in breast cancer patients under hyperglycemic conditions, leads to Cdc42 activation. Activated Cdc42 stimulates PAK1/STAT5 signaling and then activates the downstream transcriptional regulator prdm14, which maintains CSCs pluripotency and inhibits differentiation [183,184].

Beyond microRNAs that act as tumor suppressors, some microRNAs are known oncogenes. miR-548j is a pro-tumor microRNA in breast cancer, whose overexpression is related to increased invasiveness and poor prognosis [185]. It has been demonstrated that miR-548j-induced invasion is dependent on Tensin1 via Cdc42. Tensin1 can interact with the RhoGAP and DLC-1, to transform Cdc42 into inactive-bound and thus suppress invasiveness [186]. Therefore, miR-548j directly inhibits Tensin1 and protects Cdc42 in its active-bound state.

### 9.2. lncRNA

Compared to small ncRNAs, lncRNAs have relatively long nucleotide chains, which contain more protein binding sites. Metastasis associated with lung adenocarcinoma transcript-1 (MALAT1) is a lncRNA that can promote the progression of multiple tumors [187,188,189,190]. In breast cancer, miR-1 directly targets MALAT1 and Cdc42. MALAT1 can competitively bind to miR-1, which reduces the ability of miR-1 to inhibit Cdc42, ultimately increasing the level of Cdc42 and inducing cell migration and invasion to promote breast cancer metastasis [191].

## 10. Summary

The RhoGTPase family member Cdc42, which serves as a molecular switch, is essential among those normal mammary cells. The precise regulation of Cdc42 is in charge of the normal mammary gland development. However, Cdc42 is often overexpressed in breast cancer cells and predominantly acts as a pro-tumor factor accompanied with the intricate downstream signaling transduction. Cdc42 is mainly involved in the regulation of actin cytoskeleton through activating N-WASP/Arp2/3, while many of its other downstream effectors serving a number of other tasks. For instance, PAKs participates cancer cell proliferation, motility, cell death and anti-cancer drugs resistance, as well as Cdc42/Par/PKC complex affects morphogenesis and cell polarity. Cdc42 also inhibits tumor-suppressor genes to protect the breast cancer in many respects, like inhibiting *p53* to relieve its anti-proliferation, -angiogenesis and -apoptosis effects. These wide coverage functions of both Cdc42 and its effectors provide ideas for the broad-spectrum anti-cancer drug designs.

However, though the rational design of Cdc42-targeted drugs can lead to promising preclinical outcomes so far there are no drugs that target Cdc42 in clinical trials. Using the orthotopic xenograft mouse model of breast cancer is critical for Cdc42-targeted drug development and for figuring out its pharmacokinetic properties and toxicity, which are important steps in demonstrating its efficacy. Another problem emerges relating to the selectivity of many Cdc42 inhibitors; like EHop-016 and MBQ-167, which also target Rac due to the close relationship between the RhoGTPase family. Treatment strategies in the future should focus on the combination of current breast cancer therapies and Cdc42-targeted therapies, with a view toward incorporating microRNAs, to reduce metastasis and diminish drug-resistance.

## Figures and Tables

**Table 1:** The rates of Rho GTPase family and activators of Cdc42 overexpression in breast cancer.

Types		Rate of Overexpression	
		Gene Amplification/mRNA	Protein
Rho GTPase family	Rac1	>50% [7]	61.4% [8]
	Cdc42	------	42.5–56.9% [9]
The activators of Cdc42	EGFR	2–37.3% [10,11,12,13,14,15]	12.6~84.8% [10,11,14,15,16,17,18,19,20,21,22,23,24,25]

**Table 2:** Cdc42-Targeted Therapies in Breast Cancer.

Inhibitors	Therapies	Cell Lines/Tissues	Inhibitory Effects	References
GEF interaction inhibitors	EHop-016	MDA-MB-435	growth, angiogenesis, metastasis	[129]
	MBQ-167	MDA-MB-231, MCF-7 and MDA-MB-435	cell polarity, cell cycle progression, apoptosis and metastasis	[131]
		nude mice	tumor size	[131]
Nucleotide binding inhibitors	R-ketorolac	MMTV-PyMT mice	tumor progression	[140]
	MTX	PAE	cell migration.	[141]
RhoGDI modulators	secramine	Xenopus laevis cytoplasmic egg	actin polymerization	[142]
Antidiabetic drug	Metformin	MDA-MB-231	proliferation and cell migration	[146]
Biological extractions	GAEE	MDA-MB-231	cell migration	[151]
	Resveratrol	MDA-MB-231	cell migration	[122]

Abbreviations: R-ketorolac, R-enantiomer of ketorolac; MTX, mitoxantrone; PAE, porcine aortic endothelial; GAEE, Ganoderiol A-Enriched Extract.

**Table 3:** Cdc42-Related Non-Coding RNAs in Breast Cancer.

Non-Coding RNAs	RNA	Cell Lines/Tissues	Effects	Suppressor or Promoter	References
microRNAs	miR-29a	MDA-MB-453	cell cycle progression	suppressor	[156]
	miR-206	MDA-MB-231	filopodia formation and matrix degradation	suppressor	[157]
	miR-23b	MDA-MB-231, MCF-7	actin cytoskeleton	suppressor	[158]
			EMT	suppressor	[160]
			focal adhesion maturation	promoter	[160]
	miR-224	MDA-MB-231	cell invasion	suppressor	[161]
	miR-888	MCF-7	adherens junction	suppressor	[162]
	miR-424	MDA-MB-231	CSCs pluripotency	suppressor	[163]
	miR-548j	MCF-7	invasion	promoter	[165]
lncRNA	MALAT1	MDA-MB-231, MCF-7	cell migration invasion	promoter	[171]

**Abbreviations:** EMT, epithelial to mesenchymal transition; CSCs, cancer stem cells; MALAT1, Metastasis associated with lung adenocarcinoma transcript-1.
